# Supplementary material for: The Effect of Maternal Depression on Infant Attachment: A Systematic Review
Source: Int J Environ Res Public Health. 2020 Apr 14;17(8):2675. doi: 10.3390/ijerph17082675 (PMC7216154; doi:10.3390/ijerph17082675)
Supplement: Supplementary file 1 [file ijerph-17-02675-s001.pdf]

Table S1. The Quality assessment scores.

Study	Adeq-uate Sample (rev1)	Adeq-uate Sample (rev2)	Repre-Sentative (rev1)	Repre-Sentative (rev2)	Attrition (rev1)	Attrition (rev2)	Methods (rev1)	Methods (rev2)	Exper-ience (rev1)	Exper-ience (rev2)	Statistics (rev1)	Statistics (rev2)	Double Blind (rev1)	Double Blind (rev2)	Conclu-sion (rev1)	Conclu-sion (rev2)	Direct/Indirect (rev1)	Direct/Indirect (rev2)
Beeghly et al. [55]	1	1	0	0	1	1	1	1	1	1	1	1	1	1	1	1	1	1
Bergman et al. [46]	1	1	1	1	−1	−1	1	1	1	1	1	1	0	1	1	1	1	0
Biegelow et al. [56]	−1	−1	1	1	−1	−1	1	1	1	1	1	1	1	1	1	1	1	1
Carter et al. [24]	−1	−1	1	1	−1	−1	1	1	1	1	1	1	1	1	1	1	1	1
Cicchetti et al. [57]	1	1	−1	−1	1	1	1	1	0	0	1	1	0	0	1	1	1	1
Clarke−Stewart et al. [47]	1	1	1	1	1	1	1	1	1	1	1	1	1	0	1	1	1	1
De Falco et al. [48]	−1	−1	−1	−1	1	1	1	1	1	1	0	0	0	0	1	1	−1	−1
Donovan, Leavitt [58]	−1	−1	0	0	1	1	1	1	1	1	1	1	1	1	1	1	1	1
Edhbort et al. [49]	−1	−1	0	0	−1	−1	1	1	1	1	1	1	1	1	1	1	1	1
Gratz et al. [37]	−1	−1	−1	−1	1	1	1	1	1	1	1	1	1	1	0	1	−1	−1
Gravener et al. [38]	1	1	0	−1	0	0	1	1	1	1	1	1	1	1	1	1	1	1
Hayes et al. [42]	1	1	−1	−1	0	0	1	1	0	0	1	1	0	0	1	1	1	1
Hughers et al. [45]	1	1	1	1	−1	−1	1	1	1	1	1	1	1	1	1	1	1	1
Laurent, Ablow et al. [39]	−1	−1	−1	−1	0	0	1	1	1	1	1	1	1	1	1	1	1	1
Ludmer et al. [40]	1	1	−1	−1	0	0	1	1	1	1	1	1	1	1	1	1	−1	−1
Lyons-Ruth et al. [41]	1	1	−1	−1	0	0	1	1	0	0	1	1	1	1	1	1	1	1
Lyons-Ruth et al. [33]	−1	−1	1	1	0	0	1	1	1	1	1	1	1	1	1	1	0	0
McMahon et al. [52]	1	1	1	1	1	1	1	1	1	1	1	1	1	1	1	1	1	1
Measelle, Ablow et al. [53]	−1	−1	−1	−1	0	0	1	1	1	1	1	1	1	1	1	1	−1	−1
Mehler et al. [50]	0	0	−1	−1	1	1	1	1	0	0	1	1	1	1	1	1	1	1
Murray [43]	−1	−1	1	1	1	1	1	1	0	0	−1	−1	0	0	0	1	1	1
Righetti−Veltema et al. [59]	1	1	0	0	−1	−1	1	1	0	0	1	1	1	1	1	1	1	1
Sagi et al. [51]	1	1	1	1	1	1	1	1	1	1	1	1	1	1	1	1	1	1
Smith−Nielsen et al. [60]	−1	−1	−1	−1	1	1	1	1	1	1	1	1	1	1	1	1	1	1
Tarabulsy et al. [54]	1	1	−1	−1	0	0	1	1	1	1	1	1	0	0	1	1	1	1
Tharner et al. [44]	1	1	1	1	1	1	1	1	1	1	1	1	1	1	1	1	1	1
Tomlinson et al. [61]	1	1	−1	−1	1	1	1	1	0	1	1	1	1	1	1	1	1	1
Toth et al. [62]	1	1	1	1	−1	−1	1	1	1	1	1	1	1	1	1	1	1	1
Zaslow et al. [63]	1	1	1	1	1	1	1	1	0	0	1	1	0	0	1	1	1	1

Criteria used: (1) adequate sample: more than 30 participants in each group; (2) representative: sample which vary in demographic characteristics; (3) attrition rate: below 30% of initial sample; (4) validated methods: standardized methods with proper adaptation; (5) experience: the coders possessed training and experience in assessing attachment; (6) statistics: appropriately described and correct; (7) double blind: double-blind procedure in the attachment assessment; (8) conclusion: consistent with the results; (9) direct/indirect: aim of the study had direct assessment of the influence of depression on attachment style. The quality of the research was evaluated using a points system (+1 = yes, 0 = unclear, −1 = no) for all specified criteria. The maximum amount of points that a study could achieve was 9 and the minimum was 0: a possible evaluation below zero would be awarded a value of 0.
